# Dysentery

**Published:** 1890-07

**Authors:** 


### DISCUSSION.

Dr. J. W. McLaughlin had heard only the latter portion of the paper. The use of enemata, as suggested in the paper, met his approval; it is a practice with him in many cases, which he has found useful. Creolin he had thought too irritating. His rule of practice is, to be governed in prescribing by the cause of the disease. Frequently an attack of dysentery results from indigestion; in those cases where indigested food remains in the canal, his rule is, first to evacuate the bowels, either by enema or by a gentle saline, and allay the irritation and pain; then correct the fault in the digestion. If the disease is catarrhal, etc., if the discharges are mucous and attended with much tenesmus, he uses opiates. Occasionally the disease, especially in children, may be of microbic causation, fermentative. He has found naphthaline servicable here, in conjunction with enemata, if the bowels are not empty. Had recently seen a child with this variety of dysentery. He gave West's mixture with oil, followed by four or five drop doses of naphthaline. The passages were soon changed in character, and resembled "chopped meat." The naphthaline at once renders the alimentary canal antiseptic. He has found Squib's mixture excellent in allaying the pain, especially the tormina, or griping about the navel; or Squib's mixture and bismuth in cases of long standing, or subacute.

Dr. Gregg thought more importance should be attached to the diet in dysentery. The digestion being deranged, he has often found it difficult to select anything to agree with the patient, and having relieved the bowels of undigested food, it is only adding fuel to fire to load them with other food to go through the same process. The safest, and he has found it the best plan, is to withhold food a reasonable length of time. Has had no experience with disinfectants in the treatment, but is partial to cold water enemas.

Dr. Atkinson said, as to washes, one of pure water, as hot as can be borne, gives great relief. In his experience, the less medicine that is given the better, as a general thing, but he has seen cases where opiates afforded great relief, and in proper doses, were harmless. He has prescribed naphthaline with benefit, also salol. He had found that the patient had no appetite, and the trouble has been to get them to eat anything; but a little later it was just the reverse, and the difficulty was to restrain them; they wanted to eat everything. Upon the whole, he has had more behefit from washing out the bowels with large enemas of warm water than from any medical treatment.

Dr. Black, (recently resident student at Charity Hospital, New Orleans,) agreed with Dr. Garwood in the main, as to his views. He has found thirst a troublesome concomitant of dysentery, and to allay it has employed, with much benefit, mucilaginous drinks. As to enemas, he has found one containing opium very beneficial in acute cases. Also prescribes salts, syrup rhubarb and laudanum. In cases of children, he thinks best not to prescribe opiates. His favorite formula for recent dysentery is—

℞ Magnes sulph. ℥i, syr. rhu. ℥i, and tinc. opi. ʒi. To spirits lavend. comp. and aquae menth. pip. ad ℥viii. Of this, a tablespoonful given at short intervals, until relief is obtained. It promptly relieves tenesmus by unloading the engorged blood vessels of the rectum. Has also employed the ipecac treatment with some success. He finds it to create nausea, and is therefore objectionable.

Dr. W. A. Morris said the Society had discussed dysentery on many occasions before, and it is impossible to exhaust the subject, or do it justice, in the short time allowed. As is well known, there are different kinds of dysentery, produced by various causes, and in their several stages, requiring different treatment. No one formula could meet the requirements in all cases, and no rule of practice could be laid down to be invariably followed. The treatment must be adapted to the case, and intelligence should guide us in selecting our remedies. He had seen many cases in which opium was useful, in fact indispensable; in others it would be positively harmful. The secretions should never be locked up, but when the bowels have been emptied, opium will quiet peristaltic action, and lessen the pain. That is a desideratum; and oil, even though guarded by opium, will increase the peristaltic action. Naphthol and salol are useful in some cases; salines in some. When the bowels are full, large enemas give relief, and he means large, not small enemas; the latter do no good. Boracic acid he had found a good addition in some cases, and in cases of very severe tenesmus, cocaine has been introduced in the lower canal. He has seen cases of dysentery in which quinine was indicated, and answered a good purpose. The disease sometimes assumes a typhoid condition, and develops so many complications as to tax our resources. As to the use of cold water enemas, as suggeseed by one of the speakers, he had found it harmful,—it increases the tenesmus.

Dr. Garwood said it is singular how the experience of physicians would differ; that he had derived great benefit from cold injections, even ice cold, and that in the case of children it was especially grateful. He had given an ice cold enema to a child suffering with tenesmus, and it quieted him, and the child would drop off to sleep.

Dr. Cunningham said that the ground had been so fully covered by those who had spoken, that he had little or nothing to add from his own experience. The subject is particularly interesting to him, as his two children are very subject to attacks of dysentery. He has found opium, in some shape, necessary in a majority of cases, and with children, he prefers syr. morphia. This he gives with salts and syr. rhubarb. He thinks it answers all the indications, unloads the bowels and the engorged blood vessels of tne rectum, and relieves the straining, producing quiet and sleep.

Dr. Hill is partial to the old saline treatment, salts and laudanum.

Dr. Maxwell lives in a malarial district. He found that quinine is necessary in most cases. He usually combines it with small doses of gray powder and large doses of bismuth, and finds that this gives relief when other remedies have failed.

Dr. Fannie Leake found that the stools were increased by allowing the child drink. She is in the habit of rendering the water alkaline by pouring it over a little wood ashes. This allays the thirst. A favorite prescription with her is bismuth, carbolic acid, gum acacia and mint water.

Dr. R. P. Talley was surprised at the lack of enthusiasm on the part of the Society in discussing a subject of so much importance; he was reminded of a funeral. He does not approve or condemn any body's treatment, but will give a part of his own experience. He thinks we make a great mistake in hurrying to control or cut short the disease, like putting out a fire. We want to cure it right off. It cannot be done: it is a disease that must run a certain course, an inflammatory process which requires time to develop and decline. Who will pretend to say that it can be aborted? It is a catarrhal inflammation, and he is satisfied if, at the end of five to eight days, he has it well in hand. He recalled an epidemic which prevailed in Georgia, of such severity that even the dogs died; of one family of persons, fourteen died. The inflammation went on without check or abatement, and terminated in gangrene of the rectum, and in some cases even of the mouth, in spite of any and all remedies. Everything was tried; nothing succeeded. The epidemic ran its course, and was very fatal. About the beginning of the decline, a fellow by the name of Thompson—"Thompson with a p"—came there, and prescribed for some cases that got well. He got all the credit, and built up a big practice subsequently.

In his own practice, Dr. Talley uses the salts and laudanum, but uses it differently from the usual way. Gives ½ oz. of Epsom, or a tablespoonful Rochelle salts every hour, for four or five hours. This depletes the portal circulation and the blood vessels of the colon and rectum. After this is done, keep the patient still, flat of his back; put his bowels in splints, as it were; for he had observed that to turn over will often provoke painful peristalsis and tenesmus. He could not think of ice water as a remedy, but gives a large enema of warm water, and keeps it in the bowel; tampon the bowel, if necessary. This overcomes and paralyses the muscles of the bowel. Water is the great depurent, and is worth all the medicine in dysentery. Is down on the ipecac treatment, and criticised it. Likes McLaughlin's theory of microbes in the bowels, and the naphthol treatment. If given by the mouth, naphthol is not absorbed, but goes to the lower bowel. There it is absorbed, and acts as a disinfectant. If a man can take forty grains of calomel, and it goes through him, it acts as a local antiseptic in the lower bowel.

Dr. Talley spoke of a very fatal epidemic in Belton, some years ago. He lost four-fifths of his patients. Undertook to neutralize the poison, and found it required opium enough to poison the patient.

Dr. Morris asked Dr. Talley what "Dr." Thompson's treatment consisted of?

"Of calomel," said Dr. Talley, "and of roots he dug up in the woods, and stump water." The latter he had learned probably, from another quack that had made a great reputation for curing white swelling with this wonderful medicine(?).

The president thanked Dr. Talley for his interesting remarks, especially as he had given his own experience so fully.

[The JOURNAL is in receipt of a discussion of dysentery by the Taylor County Medical Society, and it is omitted because the same ground is covered in the above.—ED.]

